# Lumbar Interlaminar Ventral Epidural Injection Without Catheter at L5–S1 for Lumbosacral Radicular Pain: A Pilot Feasibility Study

**DOI:** 10.3390/medicina61112069

**Published:** 2025-11-20

**Authors:** Jiho Park, Seounghun Lee, Sunyeul Lee, ChaeSeong Lim, Yeojung Kim

**Affiliations:** 1 Department of Anesthesiology and Pain Medicine, Chungnam National University Sejong Hospital, Sejong 30099, Republic of Korea; jihopark@cnuh.co.kr (J.P.); anelee1982@cnuh.co.kr (S.L.); 2 Department of Anesthesiology and Pain Medicine, Chungnam National University College of Medicine, Daejeon 35015, Republic of Korea; neoquack@cnuh.co.kr (S.L.); limtwo2@cnuh.co.kr (C.L.); 3 Department of Anesthesiology and Pain Medicine, Chungnam National University Hospital, Daejeon 35015, Republic of Korea

**Keywords:** ventral epidural injection, interlaminar, catheter-free LIVEI, lumbosacral radicular pain, VAS, pilot feasibility study, contrast spread patterns

## Abstract

*Background and Objectives:* Lumbar interlaminar ventral epidural injection (LIVEI) offers a promising alternative to transforaminal epidural injection (TFEI) by enabling ventral epidural delivery while minimizing complication risks. While previous approaches often required catheter assistance, this pilot study evaluates the safety, technical feasibility, and early outcomes of a simplified LIVEI method at L5–S1 without catheter insertion. *Materials and Methods:* Twelve patients with lumbosacral radicular pain received unilateral catheter-free LIVEI at L5–S1 between October 2021 and September 2022. This small retrospective pilot cohort did not include a control group. Contrast spread patterns were evaluated fluoroscopically based on AP and lateral views. Spread was classified into three grades depending on anterior epidural distribution, cranio-caudal extent, and foraminal involvement. Visual Analog Scale (VAS) scores were assessed before and two weeks after the procedure. Spread was classified into three grades depending on anterior epidural distribution, cranio-caudal extent, and foraminal involvement. *Results:* Fluoroscopic images confirmed ventral epidural spread in all patients, with 75% showing foraminal extension and 67% demonstrating cranio-caudal spread over two or more levels. Baseline VAS scores averaged 6.5 ± 1.0, decreasing to 3.42 ± 1.31 two weeks post-procedure (*p* < 0.0001), with a mean reduction of 3.08 ± 1.00. No adverse events or complications were observed. *Conclusions:* Catheter-free LIVEI at the L5–S1 level demonstrated consistent anterior and multi-level ventral epidural contrast distribution on fluoroscopy, supporting the technical feasibility of this approach. In addition to this radiographic validation, patients achieved clinically meaningful pain relief with excellent tolerability. Further confirmation through larger-scale controlled studies is warranted to validate long-term clinical effectiveness.

## 1. Introduction

Lumbosacral radicular pain is common and often disabling. Epidural steroid injections (ESIs), including caudal, interlaminar, and transforaminal approaches, remain central to non-operative management [1,2,3,4]. Transforaminal epidural injection (TFEI) enables targeted delivery toward the symptomatic root sleeve [5]; however, clinical concern remains regarding inadvertent arterial entry within the foramen, intravascular injection, and rare but catastrophic ischemic events [6,7,8,9]. Comparative reports also suggest a higher overall procedure-related complication burden with TFEI compared to interlaminar techniques [10,11].

To reduce these risks, several modified interlaminar trajectories (e.g., paramedian, parasagittal) have been introduced to direct injectate more ventrally than the traditional midline posterior interlaminar route [12,13,14,15,16,17]. Lumbar interlaminar ventral epidural injection (LIVEI) was proposed as a conceptual advancement that attempts to place injectate directly in front of the dural sac by adjusting the needle path. Many of the initial LIVEI studies used a catheter to fine-tune positioning and ensure adequate anterior (ventral) spread [18].

The prior literature has shown that the pattern of epidural contrast distribution is not merely an imaging finding but can have clinical implications. Several studies have suggested that when injectate reaches the anterior (ventral) epidural space and extends along the neural foramen toward the symptomatic root sleeve, pain relief tends to be greater compared with injections in which spread remains posterior or limited [19,20,21]. Although the strength of this association has varied across studies, the accumulating evidence indicates that targeted ventral/foraminal delivery may represent an important mechanistic determinant of therapeutic response after epidural steroid injection. Consequently, techniques that reliably achieve anterior epidural spread deserve further investigation.

However, catheter-assisted LIVEI introduces practical drawbacks: longer procedure time, technical complexity, catheter-related issues (e.g., kinking or misdirection), and higher cost [22,23,24]. These limitations hinder routine adoption, particularly in high-volume outpatient settings. In contrast, catheter-free LIVEI techniques remain underexplored in the literature, and it is unknown whether they can reliably reproduce ventral epidural delivery without catheter guidance.

Therefore, this pilot study evaluated the technical feasibility and safety of catheter-free LIVEI at L5–S1, an interspace offering a relatively wide interlaminar window. In addition, fluoroscopic contrast-spread patterns and short-term analgesic response were analyzed to determine whether a catheter-free technique can consistently achieve anterior epidural spread comparable to catheter-assisted methods. We hypothesized that catheter-free LIVEI would reliably achieve ventral epidural contrast spread and could also yield meaningful short-term pain reduction.

## 2. Materials and Methods

### 2.1. Study Design and Participants

This single-center, retrospective pilot study included patients who underwent lumbar interlaminar ventral epidural injection (LIVEI) without catheter assistance at the L5–S1 level between October 2021 and September 2022. The study was performed in accordance with the principles of the Declaration of Helsinki and was approved by the Institutional Review Board of Chungnam National University Sejong Hospital (IRB No. CNUSH 2021-10-004). Given the retrospective nature and anonymized data collection, the IRB waived the requirement for written informed consent.

### 2.2. Patient Selection and Clinical Evaluation

Eligible patients were adults (>18 years) with unilateral lumbosacral radicular pain caused by lumbar disc herniation or foraminal stenosis, confirmed by MRI or CT, and refractory to at least 4 weeks of conservative therapy, including oral medication and physiotherapy.

Exclusion criteria were (1) prior lumbar surgery at the target level; (2) severe spinal deformity; (3) coagulopathy or ongoing anticoagulation therapy; (4) allergy to contrast agents or local anesthetics; (5) uncontrolled diabetes or infection; and (6) incomplete clinical or imaging data.

Fluoroscopic assessment and grading of contrast spread were performed immediately after the procedure using AP and lateral views, and spread patterns were classified according to anterior(ventral) epidural distribution, cranio-caudal coverage, and foraminal involvement. Clinical evaluation included pain severity using the Visual Analog Scale (VAS, 0–10) before the procedure and at the 2-week follow-up.

**Figure 1:**
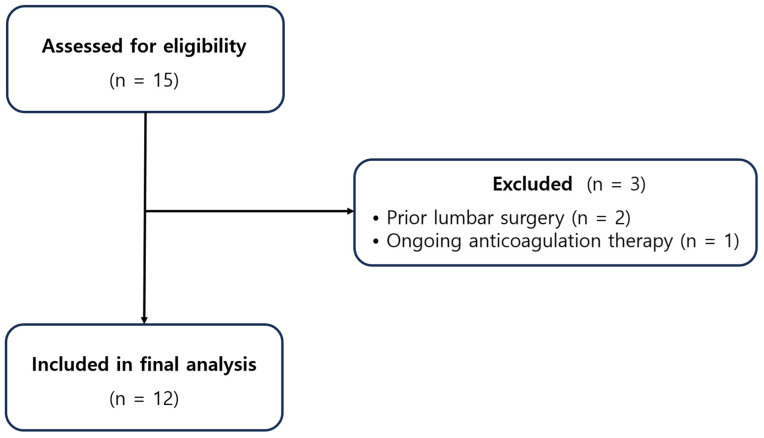
STROBE flow diagram of patient screening, exclusion, and inclusion for the final cohort.

A STROBE-style flow diagram illustrating patient screening, exclusion, and inclusion is provided (Figure 1).

### 2.3. Procedural Technique for Catheter-Free LIVEI at L5–S1

All procedures were performed by a single fellowship-trained pain specialist with more than 10 years of experience in fluoroscopy-guided spinal injections. Patients were positioned prone with a small pillow under the abdomen to reduce lumbar lordosis.

Step 1. Fluoroscopic target identification (AP)

**Figure 2:**
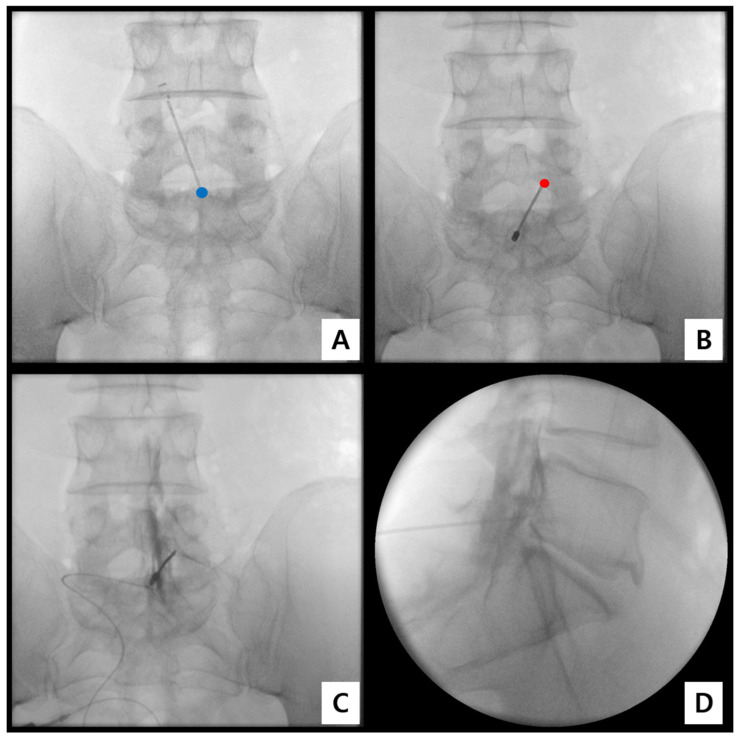
Representative fluoroscopic images during catheter-free LIVEI at the L5–S1 level. (**A**) AP view showing the interlaminar access trajectory (blue dot = insertion point). (**B**) AP view confirming the intended target vector toward the ventral epidural space (red dot = target point). (**C**) AP view during contrast injection to assess anterior (ventral) epidural spread. (**D**) Lateral view clearly demonstrating ventral epidural contrast distribution.

Under C-arm fluoroscopy (Arcadis Varic, Siemens Healthineers, Erlangen, Germany), the L5–S1 interlaminar space was visualized on an anteroposterior (AP) view with a 5–10° caudal tilt (Figure 2A).

Step 2. Midline entry and bone docking

Following aseptic preparation and local infiltration with 1% lidocaine (Huons Co., Ltd., Seongnam, Republic of Korea), an 18-gauge Tuohy needle (B. Braun Melsungen AG, Melsungen, Germany) was inserted at the midline just cephalad to the S1 spinous process and advanced cephalad toward the lower laminar border of L5, maintaining a 10–15° cephalad angle (Figure 2B).

Step 3. Interlaminar epidural access confirmation

Once bone contact was achieved, the needle was slightly redirected medially to enter the interlaminar window. Epidural space entry was confirmed using the loss-of-resistance (LOR) technique with saline, while simultaneously observing real-time AP and lateral fluoroscopy to verify consistent epidural depth. In addition, a minimal amount of Iopamiro-300 (Iopamidol 300 mg/mL; Bracco Imaging S.p.A., Milan, Italy) ≤ 0.5 mL was injected to confirm epidural placement and to exclude intrathecal or intravascular spread before further ventral advancement.

Step 4. Controlled ventral advancement (“saline cushion” technique)

**Figure 3:**
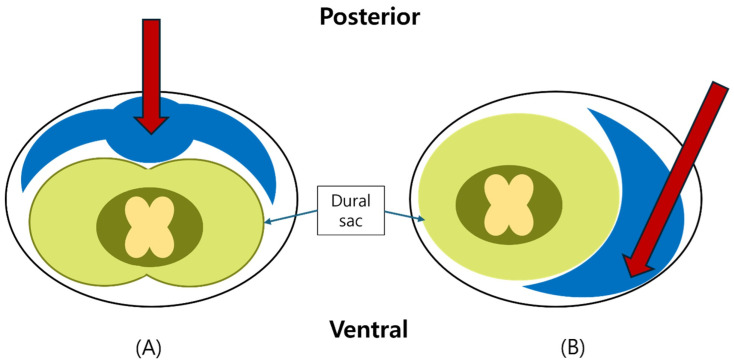
Schematic comparison of conventional posterior interlaminar epidural injection (**A**) versus lumbar interlaminar ventral epidural injection (LIVEI) (**B**). LIVEI facilitates anterior epidural distribution, allowing closer contact with the ventral epidural space and the nerve root sleeves. Blue areas indicate the injected solution, and red arrows denote the needle direction.

To prevent dural puncture, the needle bevel was oriented superolaterally and ventrally. Gentle saline injection (0.5–1 mL/s) was maintained to create a “saline cushion,” enabling subtle displacement of the dural sac to guide ventral advancement without resistance [25,26] (Figure 3).

Step 5. Fluoroscopic confirmation of ventral epidural positioning

After confirmation of epidural entry, 1–2 mL of Iopamiro-300 (Iopamidol 300 mg/mL; Bracco Imaging S.p.A., Milan, Italy) was injected under live fluoroscopy. Correct ventral placement was verified by anterior epidural contrast spread on the lateral view, along with the absence of intrathecal or vascular filling (Figure 3).

Step 6. Final injectate delivery

Once correct placement was confirmed, 4 mL of injectate—3 mL of 0.2% ropivacaine (Mitsubishi Tanabe Pharma Korea Co., Ltd., Seoul, Republic of Korea) plus 1 mL dexamethasone 5 mg/mL (Yuhan Corporation, Seoul, Republic of Korea)—was delivered slowly over 30 s. All patients were monitored for 30 min post-procedure.

The typical procedure duration from needle insertion to final injectate delivery was generally completed within 10 min.

### 2.4. Fluoroscopic Assessment and Grading of Spread

Contrast spread patterns were independently reviewed by two pain physicians blinded to clinical outcomes. Discrepancies were resolved by consensus. Spread was graded as follows [17,27]:

- Grade 3: Anterior (ventral) epidural spread with foraminal extension at two or more levels;
- Grade 2: Anterior spread with limited foraminal or cranio-caudal extent;
- Grade 1: Posterior-only or failed anterior spread.

**Figure 4:**
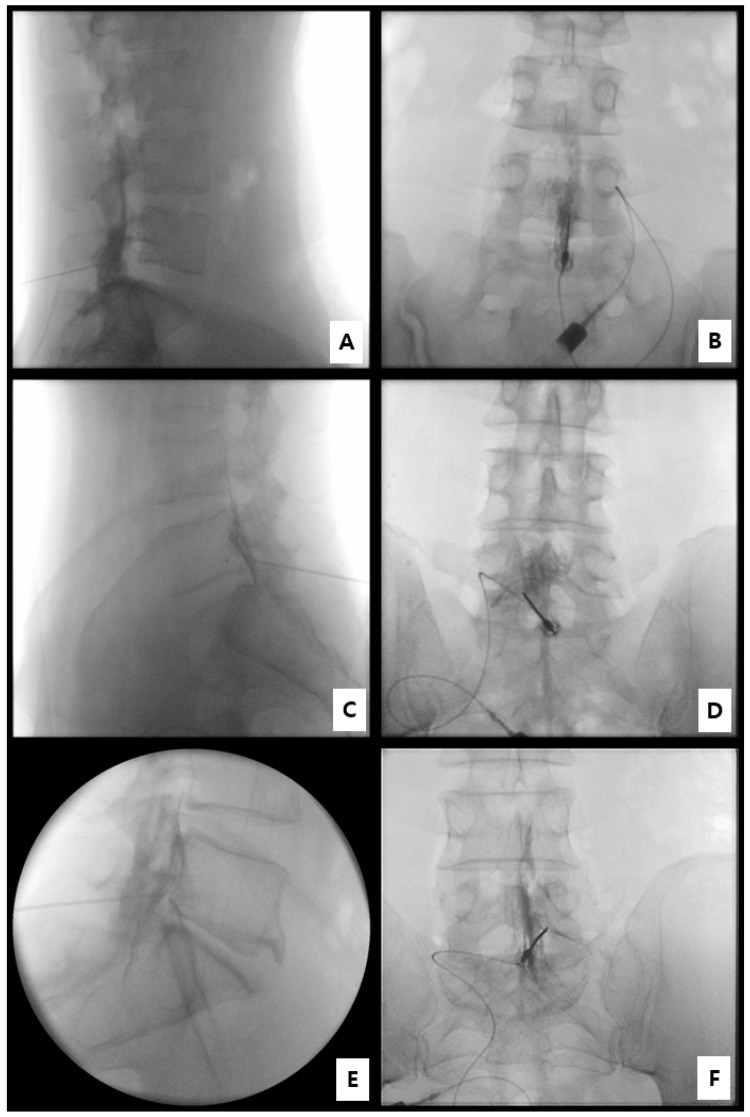
Representative fluoroscopic grading examples of contrast spread patterns. (**A**,**B**) Grade 1: Posterior-only or failed anterior spread (lateral and AP views). (**C**,**D**) Grade 2: Anterior epidural spread with limited foraminal or cranio-caudal extent (lateral and AP views). (**E**,**F**) Grade 3: Anterior (ventral) epidural spread with foraminal extension involving ≥ 2 levels (lateral and AP views).

Additional parameters recorded included foraminal involvement, the number of cranio-caudal levels, and the presence of ventral spread on both AP and lateral images. Representative AP and lateral fluoroscopic examples for Grades 1–3 are presented in Figure 4. Inter-rater agreement for spread grading was perfect across all cases (Cohen’s κ = 1.00).

### 2.5. Statistical Analysis

All statistical analyses were performed using descriptive and inferential methods. Continuous variables are presented as mean ± standard deviation (SD), and categorical variables as counts and percentages. Pain intensity, measured by the Visual Analog Scale (VAS), was compared between baseline and the 2-week follow-up using a paired *t*-test. The mean difference, SD, and 95% confidence interval (CI) were calculated.

The effect size for paired data (Cohen’s dz) was obtained by dividing the mean difference by the standard deviation of the paired differences. Clinical responders were defined as follows: (1) absolute responders—those with a ≥2-point reduction in VAS, and (2) relative responders—those with a ≥50% reduction from baseline.

All calculations were verified using an independent computational tool to ensure accuracy. A two-tailed *p* value < 0.05 was considered statistically significant.

Statistical analyses were performed using R version 4.5.2 (R Foundation for Statistical Computing, Vienna, Austria).

During the preparation of this manuscript, the authors used ChatGPT (GPT-5; OpenAI, San Francisco, CA, USA) to assist with language editing and figure caption drafting. The authors reviewed and edited the output and take full responsibility for the content of this publication.

## 3. Results

### 3.1. Baseline Characteristics

A total of 15 patients were screened, and 12 were included in the final analysis. Twelve patients (9 men, 3 women) were analyzed. Mean age was 54.17 ± 15.48 years (range 32–78). All procedures were performed at the L5–S1 level (12/12, 100%), with equal laterality (6 left, 6 right). The mean baseline VAS was 6.50 ± 1.00 (Table 1).

No patients had prior lumbar surgery, and all tolerated the procedure without acute adverse events.

### 3.2. Fluoroscopic Findings

Fluoroscopic analysis demonstrated ventral (epidural anterior) contrast spread in all 12 patients (100%). On lateral views, the contrast was seen forming a distinct crescent-shaped radiopaque layer along the posterior vertebral body margin and anterior to the dural sac, indicating true ventral epidural placement rather than posterior dispersion. On anteroposterior (AP) views, the contrast extended symmetrically from the midline toward the ipsilateral neural foramen, sometimes crossing the midline to the contralateral side in cases with more abundant spread (typically Grade 3 patterns).

In quantitative terms, 9 patients (75.0%) showed foraminal extension, defined as contrast filling the medial or lateral portion of the neural foramen adjacent to the affected nerve root. This included all Grade 3 cases (n = 8) and one Grade 2 case with limited foraminal involvement. Cranio-caudal spread over two or more intervertebral levels was observed in 8 patients (66.7%), most commonly reaching from L5–S1 to L4–L5 superiorly or to S1–S2 inferiorly.

Spread grades were classified as Grade 3 in 8 patients (66.7%), Grade 2 in 4 (33.3%), and none as Grade 1 (Table 2). Grade 3 cases consistently showed broad ventral dispersion with foraminal filling and bidirectional cranio-caudal flow, whereas Grade 2 patterns showed anterior distribution limited to one adjacent level without clear foraminal extension. No posterior-only (Grade 1) patterns were observed, underscoring the technical reliability of ventral targeting at L5–S1 using this method.

Notably, ventral contrast spread was achieved without evidence of intravascular opacification or subarachnoid diffusion, confirming that the needle position was safely within the epidural space. No leakage to paraspinal soft tissues or facet joints was seen.

### 3.3. Pain Reduction

At 2 weeks, mean VAS decreased significantly to 3.42 ± 1.31, yielding a mean improvement of 3.08 ± 0.90 points (95% CI 2.51–3.66; paired t = 11.86; *p* = 1.31 × 10^−7^). Cohen’s dz effect size was 3.42, indicating a very large treatment effect.

**Figure 5:**
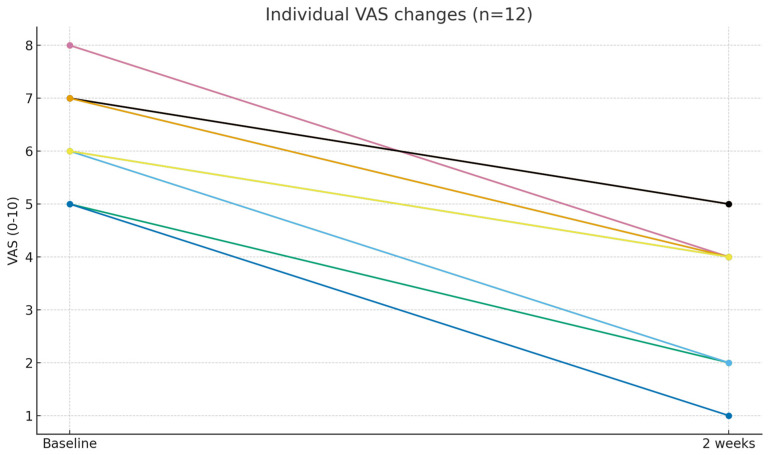
Individual VAS changes from baseline to 2 weeks after catheter-free LIVEI at L5–S1 (n = 12).

**Figure 6:**
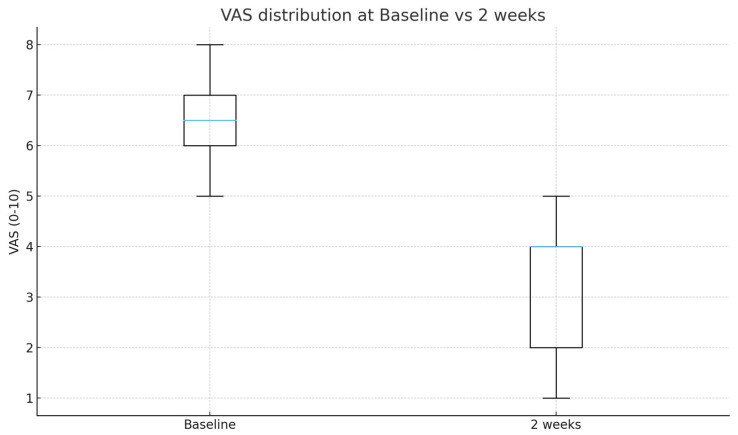
Box plot of VAS distributions at baseline versus 2 weeks (paired comparison).

All patients (100%, 12/12) achieved at least a 2-point improvement (absolute responders), and 6 patients (50%) achieved ≥50% relative pain reduction (relative responders). Individual patient trends are illustrated in Figure 5, and overall distribution changes in Figure 6. Given the small, uncontrolled cohort and short-term follow-up, these findings should be interpreted as exploratory and hypothesis-generating rather than definitive evidence of clinical efficacy.

### 3.4. Safety Outcomes

No patient experienced inadvertent intrathecal or intravascular injection, dural puncture, new neurologic deficit, or infectious complication during or after the procedure. All procedures were completed successfully on the first attempt without catheter use, and no delayed adverse events were reported within the 2-week follow-up period. No systemic steroid-related side effects (e.g., flushing, insomnia) or hemodynamic instability occurred.

## 4. Discussion

This pilot study shows that catheter-free lumbar interlaminar ventral epidural injection (LIVEI) at the L5–S1 level consistently achieves anterior epidural contrast spread. Importantly, the contrast opacified the perineural sleeve and traced the course of the exiting nerve, suggesting effective medication delivery to the pathologic site. The fluoroscopic patterns were highly reproducible across patients and consistent with previous reports of catheter-assisted LIVEI [7,14,15], suggesting that a catheter-free approach can equally achieve anterior epidural distribution when performed at L5–S1 with proper needle orientation and saline hydro-dissection. Taken together, these findings suggest that catheter-free LIVEI may represent a technically simplified and clinically meaningful approach that could serve as an alternative to transforaminal epidural injection (TFEI), particularly when direct foraminal access is challenging.

Epidural injections mainly use caudal, interlaminar, and TFEI approaches [1]. The transforaminal approach is considered more effective than the interlaminar and caudal approaches because it allows drug delivery closer to the affected nerve root [2]. The pathology likely exists around the disc and nerve root and is located in the ventral epidural space. However, numerous studies have reported significant risks such as intradiscal injection, radicular artery injection or trauma, spinal cord infarction, and paraplegia associated with TFEI [6,7,11]. The Adamkiewicz artery was located in the superior part of the foramen in 97% of the patients examined in this study [8]. As alternatives, the parasagittal approach and modified paramedian approach were reported.

Kennedy et al. published a study comparing the contrast flow patterns between the parasagittal and transforaminal approaches. In patients who underwent the parasagittal approach, spread to the ventral epidural space was observed in 100% of patients and in 75% of patients who underwent TFEI [8]. Kim et al. compared a modified interlaminar approach with the TFEI [14]. Clinical outcomes were similar between the two approaches. However, the success rate of access to the ventral epidural space was significantly higher using the modified interlaminar approach in cases with foraminal stenosis. In a study by Choi et al. comparing contrast flow in the modified paramedian interlaminar approach and TFEI in cervical epidural injection, they reported that the spread into the ventral epidural space was greater in the modified paramedian interlaminar approach than in TFEI [17].

**Figure 7:**
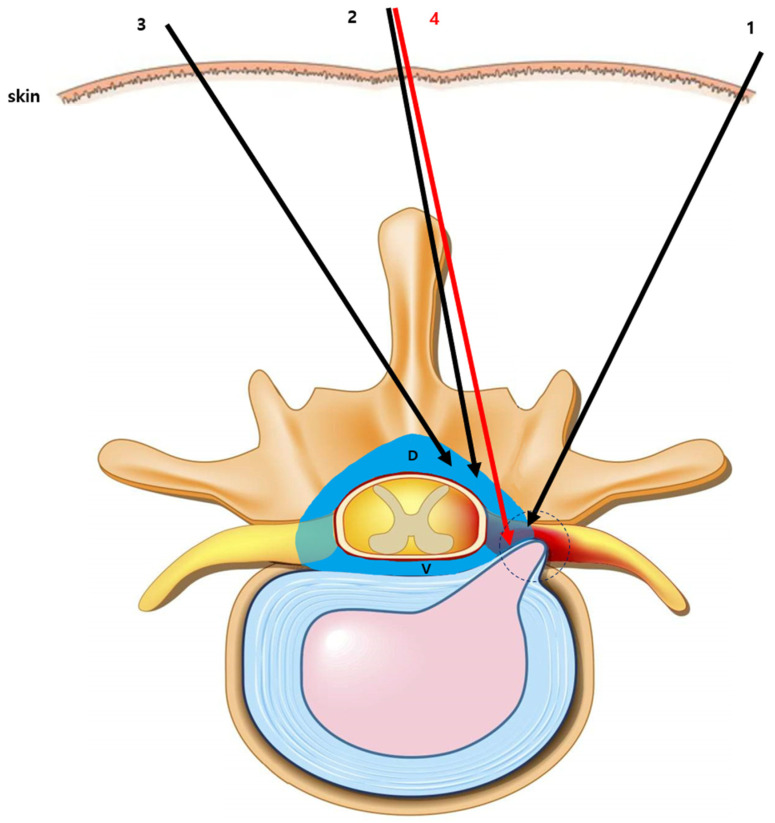
Schematic diagram showing various approaches to epidural injections. 1. TFEI. 2. Modified interlaminar approach. 3. Modified paramedian interlaminar approach. 4. LIVEI.

Approaches such as the modified paramedian interlaminar technique are difficult to perform from the contralateral side owing to spinous process interference and are methodologically complex. However, LIVEI can be easily performed using a midline approach. In addition, these alternative methods spread most of the drug to the dorsal epidural space and only a portion to the ventral epidural space. LIVEI achieved greater ventral distribution of the injectate, which may translate into improved clinical efficacy (Figure 7). In addition, LIVEI can treat multiple ipsilateral and contralateral levels simultaneously; therefore, it may offer clinical advantages compared with TFEI.

Prior studies utilizing catheter-assisted LIVEI reported favorable ventral spread and safety outcomes, and our findings indicate that a catheter may not be necessary to achieve reliable anterior distribution [18]. Furthermore, the L5–S1 interlaminar space provides a wider epidural target, lowering the risk of dural puncture and facilitating technical success.

Although effective, catheter-assisted LIVEI remains constrained by procedural complexity, longer preparation time, and catheter-related risks such as kinking, migration, or infection. These issues hinder the widespread application of injections in busy outpatient environments, highlighting the need for a simpler technique. Indeed, epidural catheterization is not risk-free—serious complications such as epidural hematoma, abscess formation, and neurological injury have been reported even in non-surgical pain procedures [22]. Moreover, catheter migration or malposition can result in unpredictable drug spread and reduced analgesic efficacy, as highlighted in a recent systematic review of epidural catheter management [23]. Likewise, catheter-based epidural injections in the cervical and thoracic spine, although technically feasible, have shown a measurable rate of procedural complications in retrospective safety analyses [24]. In this context, catheter-free LIVEI may be a simpler and potentially safer alternative by eliminating catheter-related mechanical variables while still maintaining effective ventral epidural delivery. However, catheter-free approaches remain underexplored in the literature, and their reproducibility in achieving consistent ventral epidural spread without catheter guidance requires further validation.

In addition, the technical modification of orienting the Tuohy needle bevel superolaterally and advancing with continuous saline injection allowed safe confirmation of epidural entry by subtle dural sac movement, thereby minimizing the risk of dural puncture (Figure 3) [25,26]. This maneuver enhances procedural safety and may be particularly valuable in patients with altered anatomy or prior lumbar surgery.

The consistent ventral spread pattern observed in this study supports the anatomical rationale of LIVEI. Approximately 75% of cases showed foraminal involvement, which is crucial for effective radicular pain relief. These results are in line with previous reports demonstrating that ventral targeting correlates with improved analgesic outcomes.

Beyond its procedural advantages, the catheter-free LIVEI technique has several potential clinical and research applications. In practice, it could serve as a safer and time-efficient alternative to TFEI for patients with foraminal stenosis, vascular anomalies, or prior lumbar surgery, where direct foraminal access is risky or technically limited. Because it requires no catheter and can be performed using standard fluoroscopic equipment, it may be particularly suitable for outpatient pain clinics or situations where procedural simplicity and reduced radiation exposure are desired.

From a research perspective, further prospective, randomized controlled trials are warranted to confirm these pilot findings, comparing catheter-free LIVEI directly with both TFEI and catheter-assisted LIVEI in terms of ventral contrast spread, pain reduction, and functional improvement. Longer-term studies should also assess the duration of analgesic effect, recurrence rate, and patient-reported outcomes. Moreover, future investigations could explore the use of ultrasound guidance, 3D fluoroscopy, or MRI-based epidurography to enhance procedural precision and visualize injectate dynamics in real time. Finally, the catheter-free ventral epidural approach may provide a versatile platform for targeted drug or biologic delivery, such as regenerative injectates (platelet-rich plasma, stem cell preparations, or anti-inflammatory biologics) aimed at discogenic or radicular pain. Expanding the LIVEI concept toward such minimally invasive regenerative interventions could open new therapeutic avenues, bridging interventional pain management and regenerative medicine.

Nevertheless, these results should be interpreted within the context of a small exploratory pilot feasibility study, and caution is warranted when generalizing these findings. Several important points must be emphasized. First, although catheter-free LIVEI may offer a safer alternative to TFEI by avoiding the foraminal vascular zone, this interpretation should be considered preliminary rather than definitive. Second, this study evaluated only 2-week pain outcomes and did not collect validated functional measures (e.g., ODI, PGIC); therefore, the clinical significance beyond the short term remains uncertain. Third, the present report represents a small pilot series, and the findings should not be generalized to broader patient populations or other lumbar levels. Future studies should include prospective comparative trials directly comparing catheter-free LIVEI with both TFEI and catheter-assisted LIVEI, incorporating longer-term follow-up and standardized functional outcomes, to more definitively define the clinical role of this technique. Taken together, these limitations should be explicitly acknowledged when interpreting the present findings.

## 5. Conclusions

Catheter-free lumbar interlaminar ventral epidural injection (LIVEI) is a technically feasible interventional technique that achieved reliable ventral epidural distribution without catheter assistance in this study. This simplified procedure may serve as a practical and potentially safer alternative to transforaminal epidural injection (TFEI) for patients with lumbosacral radicular pain, particularly when foraminal access is difficult or risky. Future randomized controlled trials with larger cohorts and longer-term follow-up are warranted to confirm the clinical efficacy of this technique, define optimal patient selection criteria, and more clearly establish its role in image-guided spinal interventions.

## Figures and Tables

**Table 1:** Baseline characteristics (n = 12).

Variable	Value
Age, years	54.17 ± 15.48
Sex, n (%)	Male 9 (75.0), Female 3 (25.0)
Injection level, n (%)	L5–S1, 12 (100)
Affected side, n (%)	Left 6 (50.0), Right 6 (50.0)
Baseline VAS	6.50 ± 1.00

**Table 2:** Summary of contrast spread patterns in 12 patients undergoing catheter-free LIVEI at the L5–S1 level. (n = 12).

Contrast Spread Grade	Definition	N (%)
Grade 3	Anterior (ventral) spread with foraminal extension involving ≥ 2 levels	8 (66.7%)
Grade 2	Anterior (ventral) spread with limited foraminal involvement	4 (33.3%)
Grade 1	Posterior-only or absence of anterior (ventral) spread	0 (0%)
Total	Ventral epidural spread confirmed	12 (100%)

## Data Availability

The data presented in this study are available on request from the corresponding author. The data are not publicly available due to privacy restrictions.

## Abbreviations

The following abbreviations are used in this manuscript:

LIVEI	Lumbar interlaminar ventral epidural injection
TFEI	Transforaminal epidural injection
VAS	Visual Analog Scale
IRB	Institutional Review Board

## References

[1] Abdi S., Datta S., Trescot A.M., Schultz D.M., Adlaka R., Atluri S.L., Smith H.S., Manchikanti L. (2007). Epidural steroids in the management of chronic spinal pain: A systematic review. Pain Physician.

[2] Airaksinen O., Brox J.I., Cedraschi C., Hildebrandt J., Klaber-Moffett J., Kovacs F., Mannion A.F., Reis S., Staal J.B., Ursin H. (2006). Chapter 4. European guidelines for the management of chronic nonspecific low back pain. Eur. Spine J..

[3] Benyamin R.M., Wang V.C., Vallejo R., Singh V., Helm S. (2012). A systematic evaluation of thoracic interlaminar epidural injections. Pain Physician.

[4] Pinto R.Z., Maher C.G., Ferreira M.L., Hancock M., Oliveira V.C., McLachlan A.J., Koes B., Ferreira P.H. (2012). Epidural corticosteroid injections in the management of sciatica: A systematic review and meta-analysis. Ann. Intern. Med..

[5] Manchikanti L., Buenaventura R.M., Manchikanti K.N., Ruan X., Gupta S., Smith H.S., Christo P.J., Ward S.P. (2012). Effectiveness of therapeutic lumbar transforaminal epidural steroid injections in managing lumbar spinal pain. Pain Physician.

[6] Atluri S., Glaser S.E., Shah R.V., Sudarshan G. (2013). Needle position analysis in cases of paralysis from transforaminal epidurals: Consider alternative approaches to traditional technique. Pain Physician.

[7] Candido K.D., Katz J.A., Chinthagada M., McCarthy R.A., Knezevic N.N. (2010). Incidence of intradiscal injection during lumbar fluoroscopically guided transforaminal and interlaminar epidural steroid injections. Anesth. Analg..

[8] Kennedy D.J., Dreyfuss P., Aprill C.N., Bogduk N. (2009). Paraplegia following image-guided transforaminal lumbar spine epidural steroid injection: Two case reports. Pain Med..

[9] Mandell J.C., Czuczman G.J., Gaviola G.C., Ghazikhanian V., Cho C.H. (2017). The lumbar neural foramen and transforaminal epidural steroid injections: An anatomic review with key safety considerations in planning the percutaneous approach. AJR Am. J. Roentgenol..

[10] Wadhwa H., Rohde M., Koltsov J.C.B., Cabell A., Smuck M., Hu S.S., Kleimeyer J.P. (2025). Incidence and risk factors for complications following cervical epidural steroid injections. Spine J..

[11] Rozin L., Rozin R., Koehler S.A., Shakir A., Ladham S., Barmada M., Dominick J., Wecht C.H. (2003). Death during transforaminal epidural steroid nerve root block (C7) due to perforation of the left vertebral artery. Am. J. Forensic Med. Pathol..

[12] Ghai B., Vadaje K.S., Wig J., Dhillon M.S. (2013). Lateral parasagittal versus midline interlaminar lumbar epidural steroid injection for management of low back pain with lumbosacral radicular pain: A double-blind, randomized study. Anesth. Analg..

[13] Hashemi M., Mofrad M.K., Mohajerani S.A., Kazemi S.M., Radpey B., Zali A. (2015). Anatomical flow pattern of contrast in lumbar epidural space: A human study with a midline vs. parasagittal interlaminar approach under fluoroscopy. Pain Physician.

[14] Kim E.D., Roh M.S., Park J.J., Jo D. (2016). Comparison of the ventral epidural spreading in modified interlaminar approach and transforaminal approach: A randomized, double-blind study. Pain Med..

[15] Manchikanti L., Knezevic N.N., Navani A., Christo P.J., Limerick G., Calodney A.K., Grider J., Harned M.E., Cintron L., Gharibo C.G. (2021). Epidural interventions in the management of chronic spinal pain: American Society of Interventional Pain Physicians (ASIPP) comprehensive evidence-based guidelines. Pain Physician.

[16] Gebrekristos B., Turcu R., Kotler D., Gureck A.E., Meleger A.L. (2023). An update on technical and safety practice patterns of interlaminar epidural steroid injections. Interv. Pain Med..

[17] Choi E., Nahm F.S., Lee P.B. (2015). Comparison of contrast flow and clinical effectiveness between a modified paramedian interlaminar approach and transforaminal approach in cervical epidural steroid injection. Br. J. Anaesth..

[18] Choi Y.K., Barbella J.D. (2009). Evaluation of epidurographic contrast patterns with fluoroscopically guided lumbar interlaminar ventral epidural injection. Pain Pract..

[19] Kim D., Kim S.H., Lee S., Park S.B. (2018). Fluoroscopic analysis of ventral epidural spread in parasagittal interlaminar approach compared with transforaminal epidural injection. Pain Physician.

[20] Manchikanti L., Boswell M.V., Singh V., Benyamin R.M., Fellows B., Falco F.J. (2009). Safety and complications of epidural steroids: Evaluation of intravascular injection and epidurograms. Pain Physician.

[21] Choi E., Park K.D., Lee J., Kim S., Lee M., Kim H., Yang Y., Lee D., Kwon T., Jeong C. (2022). Modified interlaminar epidural approach versus transforaminal injection in cervical radicular pain: Contrast flow comparison under fluoroscopy. Korean J Pain..

[22] Seidel R., Tietke M., Heese O., Walter U. (2021). Serious complications after epidural catheter placement: Two case reports. Local Reg. Anesth..

[23] van Kassel M.N., Hermanides J., Lirk P., Hollmann M.W. (2025). Prevention of epidural catheter migration and inflammation by tunneling: A systematic review and meta-analysis. J. Clin. Med..

[24] Hasoon J., Mahmood S., Mahmood S., Kaye A.D., Robinson C.L. (2025). Safety of catheter-based cervical epidural steroid injections: A retrospective review. Orthop. Rev..

[25] Toomey P.J. (1992). A technique to avoid dural puncture by the epidural catheter. Anaesthesia.

[26] Tseng C.H., Li A.H., Kuo-Sheng H., Wu R.S., Tan P.P. (1995). Prior epidural injection of 10 mL normal saline reduces the incidence of inadvertent venous puncture in epidural catheterization. Acta Anaesthesiol. Sin..

[27] Candido K.D., Raghavendra M.S., Chinthagada M., Badiee S., Trepashko D.W. (2008). A prospective evaluation of iodinated contrast flow patterns with fluoroscopically guided lumbar epidural steroid injections: The lateral parasagittal interlaminar versus transforaminal approach. Anesth. Analg..

